# Inhaled nitric oxide for the adjunctive therapy of severe malaria: Protocol for a randomized controlled trial

**DOI:** 10.1186/1745-6215-12-176

**Published:** 2011-07-13

**Authors:** Michael Hawkes, Robert O Opoka, Sophie Namasopo, Christopher Miller, Kevin E Thorpe, James V Lavery, Andrea L Conroy, W Conrad Liles, Chandy C John, Kevin C Kain

**Affiliations:** 1 Institute of Medical Sciences, University of Toronto, Canada; 2 Division of Infectious Diseases, Department of Pediatrics, The Hospital for Sick Children, Toronto, Canada; 3 Department of Paediatrics and Child Health, Mulago Hospital and Makerere University, Kampala, Uganda; 4 Department of Paediatrics, Jinja Regional Referral Hospital, Jinja, Uganda; 5 Department of Respiratory Medicine, Faculty of Medicine, University of British Columbia, Vancouver, Canada; 6 Dalla Lana School of Public Health, University of Toronto, Canada; 7 Applied Health Research Centre, St Michael's Hospital, Toronto, Ontario, Canada; 8 Centre for Global Health Research, The Keenan Research Centre, Li Ka Shing Knowledge Institute, St. Michael's Hospital, Toronto, Canada; 9 Department of Public Health Sciences and Joint Centre for Bioethics at the University of Toronto, Canada; 10 Department of Laboratory Medicine and Pathobiology, University of Toronto, Canada; 11 Department of Medicine, University of Toronto, Toronto, Canada; 12 Sandra A. Rotman Laboratories, McLaughlin-Rotman Centre for Global Health, Toronto, Canada; 13 McLaughlin Centre for Molecular Medicine, Toronto, Canada; 14 Tropical Disease Unit, Toronto General Hospital, Toronto, Canada; 15 Division of Global Pediatrics, Department of Pediatrics, University of Minnesota, Minnesota, USA

## Abstract

**Background:**

Severe malaria remains a major cause of global morbidity and mortality. Despite the use of potent anti-parasitic agents, the mortality rate in severe malaria remains high. Adjunctive therapies that target the underlying pathophysiology of severe malaria may further reduce morbidity and mortality. Endothelial activation plays a central role in the pathogenesis of severe malaria, of which angiopoietin-2 (Ang-2) has recently been shown to function as a key regulator. Nitric oxide (NO) is a major inhibitor of Ang-2 release from endothelium and has been shown to decrease endothelial inflammation and reduce the adhesion of parasitized erythrocytes. Low-flow inhaled nitric oxide (iNO) gas is a US FDA-approved treatment for hypoxic respiratory failure in neonates.

**Methods/Design:**

This prospective, parallel arm, randomized, placebo-controlled, blinded clinical trial compares adjunctive continuous inhaled nitric oxide at 80 ppm to placebo (both arms receiving standard anti-malarial therapy), among Ugandan children aged 1-10 years of age with severe malaria. The primary endpoint is the longitudinal change in Ang-2, an objective and quantitative biomarker of malaria severity, which will be analysed using a mixed-effects linear model. Secondary endpoints include mortality, recovery time, parasite clearance and neurocognitive sequelae.

**Discussion:**

Noteworthy aspects of this trial design include its efficient sample size supported by a computer simulation study to evaluate statistical power, meticulous attention to complex ethical issues in a cross-cultural setting, and innovative strategies for safety monitoring and blinding to treatment allocation in a resource-constrained setting in sub-Saharan Africa.

**Trial Registration:**

ClinicalTrials.gov Identifier: NCT01255215

## Background

Malaria is the leading parasitic cause of morbidity and mortality worldwide, causing an estimated 240 million clinical cases and 800,000 deaths annually [1]. Children in sub-Saharan Africa bear the greatest burden of disease, where one in every five childhood deaths is due to malaria and 25% of survivors of cerebral malaria develop long-term neurocognitive impairment [1,2]. Despite the use of highly effective anti-malarial medications, 10-30% patients with severe malaria will die [3-5], underscoring the need for adjunctive therapies that can be applied in endemic areas. To date, effective adjunctive treatments have been elusive despite numerous clinical trials [6]. New therapies, appropriate for use in endemic areas, are therefore urgently needed to address the unacceptably high residual mortality associated with severe malaria in pediatric populations.

NO is a gaseous, lipid-soluble free radical that is produced *in vivo *by the enzymatic conversion of L-arginine and molecular oxygen to L-citrulline by members of the nitric oxide synthase (NOS) family of proteins. A free radical, NO is a highly labile molecule with a half-life of several seconds that reacts with transition metal or thiol groups of numerous target proteins [7]. NO readily diffuses across cell membranes into neighbouring cells, or may produce effects distant from its site of production transported by vehicles such as low-molecular weight S-nitrosotiols, S-nitrosylated proteins including haemoglobin and albumin, and nitrosyl-metal complexes which liberate NO spontaneously or after cleavage by ectoenzymes [8]. NO regulates numerous cellular processes including cytoplasmic granule exocytosis, platelet aggregation, endothelial-cell activation, apoptosis, inflammation, chemotaxis, neurotransmission and antimicrobial defense by modulating the activity of regulatory proteins [9]. A well-recognized example is the role of NO as the endothelium-derived relaxation factor that mediates vasodilation by activating smooth muscle soluble guanylate cyclase (sGC) [10].

Evidence from a murine model suggests that reduced NO bioavailability contributes to the pathogenesis of experimental cerebral malaria [11]. "Footprint" molecules of labile nitric oxide including cGMP and nitrite were markedly decreased over the course of infection, and NO supplementation with either a NO donor (dipropylenetriamine NONOate, DPTA/NO) or NO gas provided marked protection against severe disease [11]. Data from human studies support the hypothesis of reduced bioavailable NO in severe malaria: African children with severe malaria have impaired production of NO [12], low levels of mononuclear cell iNOS expression [12], low levels of the NOS substrate arginine [13], and elevated levels of the NOS inhibitor, asymmetric dimethyl arginine [14]. Furthermore, genetic variation in NOS isoforms that affect plasma and urine levels of NO and its metabolites are common in African populations and have been shown to influence the risk of cerebral malaria [15-19]. Factors contributing to reduced bioavailable NO in malaria include scavenging of NO by free haemoglobin and superoxide anion, and reduced levels of nitrate, a NO precursor molecule [11,13,20].

The mechanism of action by which NO might improve outcomes in malaria may involve the vascular endothelium, which plays a central role in the pathogenesis of cerebral malaria. Activation of endothelial cells is characterized by increased surface expression of cellular adhesion molecules, the exocytosis of Weibel-Palade bodies (WPB), and the breakdown of intracellular tight junctions with transudation of intravascular fluid producing end organ dysfunction. In malaria, parasitized erythrocytes (PEs) adhere to the microvascular endothelium, resulting in sequestration and vascular obstruction, impaired perfusion and tissue hypoxia [21]. Autopsy studies in fatal cerebral malaria reveal sequestration of PEs in the capillaries and post-capillary venules of multiple organs [22]. NO decreases endothelial cell adhesion molecule expression, including intercellular cell adhesion molecule-1 (ICAM-1) [23,24] and has been shown to reduce the adherence of PEs to endothelial cells [25]. The release of intracellular WPB contents from endothelial cells liberates von Willebrand factor (vWF) [26,27] and angiopoietin-2 (Ang-2) [28,29] into the circulation. Interactions of vWF with the coagulation cascade may contribute to vessel obstruction and may help tether parasitized erythrocytes to endothelial cells via platelets [30]. Ang-2 acts in an autocrine and paracrine fashion to sensitize the endothelium to the effects of TNF, resulting in increased adhesion receptor expression [31]. In addition, Ang-2 antagonizes the interaction of the Tie-2 receptor with angiopoietin-1 (Ang-1), thereby promoting endothelial permeability and reducing vessel stability [32-35]. NO inhibits the exocytosis of WPB contents through S-nitrosylation of critical regulatory factors [9] and may therefore promote endothelial quiescence, reduce vascular fluid leak, and reduce end-organ damage. A clinical trial demonstrated that a strategy of NO supplementation using the NOS substrate L-arginine improved endothelial function, as measured by reactive-hyperemia-peripheral arterial tonometry, in Indonesian adults with moderately severe malaria [36].

Low-flow iNO at a concentration of 5-80 ppm is approved for use by the US FDA for the treatment of neonates with hypoxic respiratory failure, in whom it reduces requirements for extracorporeal membrane oxygenation (ECMO) and improves survival [37]. After a decade of use in clinical practice and in numerous clinical trials of iNO in critically ill older children and adults, iNO has a well-established safety profile. Pragmatic considerations, including a theoretically cheap manufacturing cost and ease of administration by mask, make NO an attractive therapeutic option for unresponsive patients in resource-limited settings.

In summary, a clinical trial of adjunctive inhaled nitric oxide (iNO) in severe malaria is warranted on the basis of firm proof of concept from animal models [11] and a clinical trial using the NO donor L-arginine [36], together with evidence of safety from clinical experience and numerous clinical trials of iNO for other conditions [38].

## Methods

### Study Design

**Figure 1:**
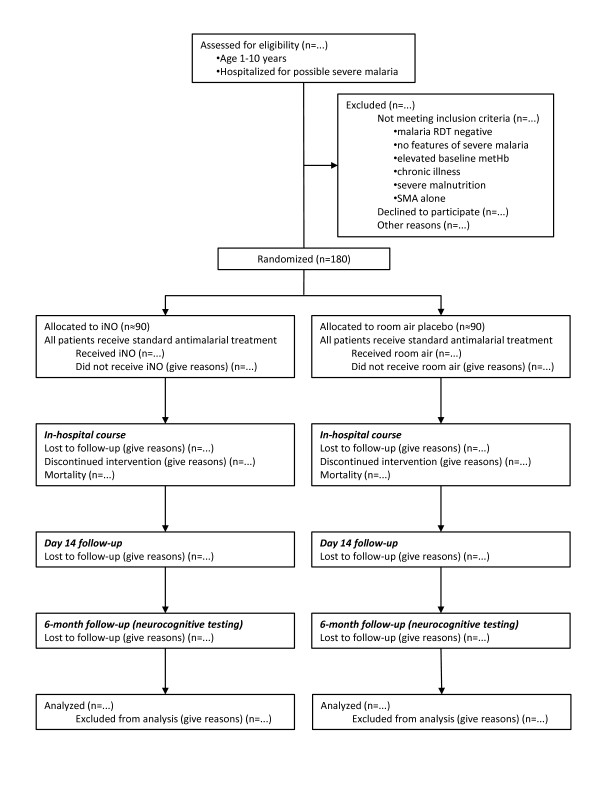
**Participant flow diagram**. The participant flow diagram illustrates randomization of 180 children with severe malaria to inhaled nitric oxide (iNO) or placebo, consistent with the Consolidated Standards of Reporting Trials (CONSORT) 2010 statement. RDT: rapid diagnostic test; metHb: methemoglobin; SMA: severe malarial anemia.

The study is a prospective, parallel arm, randomized, placebo-controlled, blinded clinical trial of adjunctive continuous inhaled nitric oxide at 80 ppm versus placebo (both arms in addition to standard anti-malarial therapy), among children aged 1-10 years of age with severe malaria. Figure 1 shows a participant flow diagram for the trial, consistent with the Consolidated Standards of Reporting Trials (CONSORT) 2010 statement [39].

### Study Objectives

The primary objective of this trial is to compare the longitudinal change in Ang-2, an objective and quantitative biochemical marker of malaria severity, among children who are randomized to receive inhaled nitric oxide in addition to standard antimalarial therapy compared to those randomized to placebo in addition to standard antimalarial therapy.

### Secondary objectives are

• To determine the efficacy of adjunctive iNO in severe malaria based on clinical parameters: mortality, recovery times, length of hospital stay.

• To assess the effect of adjunctive iNO on laboratory parameters: parasite clearance, whole blood lactate levels, and other biomarkers of malaria severity.

• To determine the efficacy of adjunctive nitric oxide in preventing neurocognitive sequelae after severe malaria.

• To assess the tolerability and safety of iNO in severe malaria.

### Study Hypotheses

The working hypothesis is that young children hospitalized with malaria will benefit from adjunctive iNO, as determined by more rapid improvement in serum Ang-2 levels. We will test this hypothesis by comparing the change in Ang-2 over the hospital admission between the two groups randomized to receive iNO or placebo (room air) using a mixed-effects linear statistical model.

The secondary hypotheses are that adjunctive iNO will reduce mortality, accelerate recovery times, and shorten length of hospital stay in severe malaria. Furthermore, we hypothesize that iNO will accelerate improvements in biomarkers of host response to severe malaria, including whole blood lactate, but will not affect parasite clearance. We hypothesize that iNO will reduce the rate of adverse neurocognitive sequelae following severe malaria. Finally, we hypothesize that adjunctive iNO will be safe and well tolerated in children treated for severe malaria.

### Eligibility criteria

Children will be eligible for the trial if they meet the following inclusion criteria:

1. Age 1-10 years

2. Positive malaria rapid diagnostic test (RDT)

3. One or more features of severe malaria: repeated seizures (two or more generalized seizures in 24 h); prostration (in children 1 year and older, the child is unable to sit unsupported or stand although was able to before the illness); impaired consciousness (Blantyre Coma Score <5 in children 1 to 4 years, GCS <14 for children ≥ 5 years); respiratory distress: age related tachypnea with sustained nasal flaring, deep breathing or subcostal retractions

4. Willing and able to complete follow up schedules for the study - 14 day and 6 months after hospital discharge.

The inclusion criteria require timely parasitological confirmation of malaria infection prior to enrolment, which poses logistical challenges at our peripheral centre with limited laboratory resources. For diagnosis, we will use commercially available immunochromatographic RDTs, complemented where possible with microscopy of peripheral smears. Despite well-recognized variability in the test performance characteristics of RDTs under field conditions, one commercially available RDT (First Response Malaria Ag Combo (pLDH/HRP2), Premier Medical Corporation Ltd., India) is highly ranked by the World Health Organisation (WHO), with a 95% detection rate even at low parasitemia and a false positive rate of 0% [40]. This RDT includes detection bands for two *P. falciparum *antigens, histidine-rich protein-2 (HRP-2) and parasite lactate dehydrogenase (pLDH), and we will require positivity for both antigens for trial inclusion. Thus, our parasitologic criterion is expected to be highly specific, in order to include only patients who are truly parasitemic.

The exclusion criteria for this study are as follows:

1. Baseline methemoglobinemia (>2%)

2. Known chronic illness: renal, cardiac, or hepatic disease, diabetes, epilepsy, cerebral palsy, or clinical AIDS

3. Severe malnutrition, defined as weight-for length or height below -3 standard deviations based on WHO reference charts, or symmetrical edema involving at least the feet [41].

4. Severe malarial anemia (SMA; Hb <50 g/L) without other signs of severe malaria.

The latter exclusion criterion was chosen because of differences in the pathophysiology of SMA (increased clearance of infected and uninfected erythrocytes, and dysregulated hematopoiesis) compared to other malaria syndromes characterised by excessive inflammation and endothelial activation. Based on its postulated mechanism of action, it is less clear that iNO would benefit children with SMA.

### Study setting

The study will be conducted at a single pediatric hospital in Jinja, Uganda. Uganda is a low-income country with a severely resource-constrained healthcare system. Malaria transmission is moderate and seasonal in Jinja, which lies on the northern shore of Lake Victoria in the central area of Uganda near the capital, Kampala. Jinja Regional Referral hospital admits at least 175 children with severe malaria annually (excluding cases of severe malarial anemia), representing over 30% of all admissions. *P. falciparum *resistance to chloroquine and sulfadoxine-pyrimethamine is widespread in the country (34% to 67%) [42].

### Treatment groups

Participants in the intervention group will receive iNO at a concentration of 80 ppm, in addition to Ugandan standard of care of severe malaria, which includes a potent anti-parasitic agent. In light of the recent AQUAMAT trial that demonstrated a mortality benefit of parenteral artesunate over quinine for the treatment of African children with severe malaria [5], artesunate will be used as the antimalarial of choice for this study. iNO will be administered continuously via non-rebreather face mask for a maximum period of 72 hours, but may be discontinued earlier if a patient recovers and no longer tolerates the face mask. The dose of iNO (80 ppm) was chosen as it is the highest approved dose by the US-FDA for use in neonates, with the greatest potential for a therapeutic effect. In pre-clinical animal studies, a dose of 80 ppm provided greater protection from experimental cerebral malaria than lower doses (Serghides, Kim, et al., unpublished data). At least five published clinical trials across different age groups and clinical conditions have safely used this dose [43-47]. With careful monitoring for dose-dependent adverse events and titration of iNO concentration accordingly, a dose of 80 ppm can safely be used in our trial.

Participants in the control group will receive room air (indistinguishable in odour and appearance from the mixture of 80 ppm iNO), in addition to Ugandan standard of care treatment for severe malaria, including parenteral artesunate. An air compressor will be used to deliver continuous flow of vehicle air in both groups.

### Randomization and blinding methods

Simple randomization will be employed, using a computer-generated randomization list. Treatment allocation will be recorded on paper and kept in sequentially numbered sealed opaque envelopes, which will be drawn for each randomized participant by an unblinded investigator who is not responsible for patient care, laboratory or data analysis. We will retain all envelopes and records for quality monitoring purposes.

In previous clinical trials using iNO, one of the design and implementation challenges was establishing the blinding procedures while titrating and monitoring concentrations of iNO, as well as anticipated dose-related increases of methemoglobin and NO_2 _concentrations [38]. The establishment of two teams, one blinded team making all clinical assessments and therapeutic decisions, and another unblinded team monitoring the delivery of the treatment gas and assessing the development of potential toxicities, allowed iNO to be delivered safely while minimizing the possibility that direct knowledge of treatment allocation would influence the care delivered to the patient [38]. Thus, an unblinded investigator not involved in patient care will be responsible for the administration, monitoring and recording of iNO, NO_2_, and methemoglobin levels. Cylinders containing NO will be attached to the ventilation circuit in all patients, but the flow of NO will be controlled according to treatment arm assignment in a manner blinded to patients, caregivers, and healthcare providers. Only the unblinded study site investigator will administer, monitor, and titrate the delivery of NO or placebo (room air), as well as monitor and record the safety parameters of methemoglobin and NO_2 _levels. Laboratory analysis for Ang-2 levels (primary outcome) and all other parameter will occur in a manner blinded to treatment allocation.

### Outcome measures

The longitudinal change in serum Ang-2 concentration over the first 72 hours of hospital admission will be the primary efficacy endpoint. Elevated Ang-2 levels are associated with poor clinical outcome in severe malaria [28,29] and Ang-2 has been used to follow disease progression and recovery in previous studies of malaria [28]. Among survivors of severe malaria, Ang-2 levels have been shown to decrease linearly during recovery at a mean rate of 2700 pg/mL per 24 h [28]. Thus, Ang-2 is an objective, quantitative marker of disease severity, validated for longitudinal follow-up of patients with malaria. Ang-2 levels will be measured longitudinally at admission (day 0), day 1, day 2 and day 3 of hospitalization. Angiopoietin-2 will be measured by enzyme-linked immunosorbent assay (ELISA) from plasma or serum samples, and is readily detectable in samples frozen for storage and later thawed [6-9]. Commercially available ELISA kits will be used (DuoSets, R&D Systems, Minneapolis, MN). A mixed-effects linear model will be used to compare the change in Ang-2 over time between treatment arms.

Secondary outcomes of the trial will include relevant clinical, laboratory and neurocognitive endpoints. We will compare the following clinical endpoints, consistent with other therapeutic trials for malaria [4,48,49]: mortality at 48 hours and 14 days after admission; recovery times (time to fever resolution, time to sit unsupported); and length of hospital stay. Parasitological outcomes including time to parasite clearance and parasite recrudescence/re-infection at 14 day follow-up will also be compared between treatment groups. Biomarkers of disease severity (in addition to Ang-2, the primary study endpoint), including whole blood lactate, will also be followed. Lactate is produced by the anaerobic metabolism of glucose in the absence of adequate tissue oxygenation, and elevated lactate levels represent a final common pathway of tissue hypoxia and decompensated shock, the forerunner of cardiovascular collapse and death. We will measure lactate as an independent biomarker of disease severity during the clinical trial. Biomarkers of endothelial activation, inflammation, and coagulopathy will also be followed as they may provide additional insight into the pathways and processes altered in cerebral malaria and modulated by iNO delivery. Finally, neurocognitive outcomes in children with severe malaria will be followed in order to determine if adjunctive iNO may have a neuroprotective effect. The overall cognitive deficit at 6 months after discharge will be assessed by performing neuropsychological tests as previously described [2].

### Duration of Study Participation

After admission to hospital for severe malaria, disease survivors will typically be discharged after a week or less. Trial participants will be administered iNO during the first 72 hours of admission (or less if a patient recovers and no longer tolerates the mask), which represents the period of highest mortality. During hospitalization, detailed data on the interim medical history will be collected, with attention to complications like coma, seizures and hypoglycemia that might affect neurocognitive outcome. After discharge, patients will return for a follow-up visit at 14 days to test for *P. falciparum *recrudescence, and at 6-months to undergo neurocognitive testing.

### Safety

NO is approved by the US FDA for the treatment of neonates with hypoxic respiratory failure, where it has been shown to improve oxygenation, decrease pulmonary hypertension, reduce the requirement for extracorporeal membrane oxygenation, and improve survival [37]. In addition, iNO is widely used in clinical practice across North America and Europe in older children and adults with respiratory failure, where it improves oxygenation but has not been shown to confer a survival benefit [50]. As adjunctive therapy, iNO is safe and well tolerated in these critically ill patient populations and a large number of randomized controlled trials have demonstrated a favourable safety profile of iNO. One meta-analysis of 12 trials including 1237 patients with acute lung injury or acute respiratory distress syndrome demonstrated that iNO is generally safe, but was associated with a statistically elevated risk of developing renal dysfunction in these critically ill adults [38]. We anticipate that our pediatric target population may be less susceptible to renal injury, particularly after exclusion of patients with underlying chronic renal disease, but we will monitor renal function in all patients enrolled in our trial with daily creatinine and urine output measurement.

Methemoglobinemia and elevated nitrogen dioxide (NO_2_) levels in the inspiratory ventilation circuit are well-recognized, dose-dependent, and reversible adverse effects of nitric oxide administration; however, at doses commonly used in clinical practice, these are not common or clinically important consequences [38]. Methemoglobinemia results from the reaction of NO with oxyhemoglobin, thereby reducing oxygen carrying capacity [51]. NO_2 _is generated from the gas phase reaction of NO with molecular oxygen, and is a known pulmonary irritant [52,53]. In previous clinical trials of iNO, methemoglobin and NO_2 _were routinely monitored and elevated levels constituted a criterion for NO dose reduction. Among neonates receiving iNO at 80 ppm, 35% developed methemoglobinemia (>7%) and 19% had elevated NO_2 _levels (>3%), requiring reduction of the iNO concentration in the ventilation circuit [44]. Similarly, in our trial, methemoglobin and NO_2 _will be monitored and the iNO dose will be titrated downward according to these defined thresholds. Furthermore, elevated baseline methemoglobinemia will be used as an exclusion criterion from study participation as a possible indicator of genetic susceptibility to methemoglobinemia.

Adverse events may occur commonly in a trial involving children with severe malaria, although the majority of events are likely due to the clinical course of the infection and not to study medications. For example, mortality among Ugandan children with severe malaria receiving standard care including potent antimalarial agents was as high as 16% among children with impaired consciousness and 21% among children with deep acidotic breathing as presenting clinical signs [42]. In order to carefully and rationally monitor the frequency of deaths in our trial for deviations from the expected baseline level, we plan to use statistical control charts. The control chart is a commonly used tool to monitor output of processes in a variety of settings, including clinical trials [54]. This method continuously follows a process outcome (e.g., patient mortality), allowing early detection of deviations from a state of "statistical control," thereby prompting a search for assignable causes. Further details of the statistical thresholds and the performance characteristics of the control chart for mortality in this trial are given in a supplementary file (Additional File 1).

Other adverse events will be monitored using pediatric toxicity tables modified from the US National Institute of Allergy and Infectious Diseases [55]. Using this comprehensive checklist of potential adverse events, investigators will grade the severity of the event and the likelihood that the event is causally associated with the study gas according to scales defined *a priori *[55,56].

In addition to the ethical oversight provided by both the Ugandan and North American institutions, an independent Data and Safety Monitoring Board (DSMB) has been convened to supervise the trial. The DSMB is composed of medical and biostatistical experts with representation from Uganda and North America who will meet periodically and as necessary to review trial progress, safety data, and indices of study quality. Severe adverse events, including all deaths in the trial, will be reported in a timely fashion to the DSMB and to the ethics boards that approved the study.

### Ethical considerations

Ethical approval has been obtained from the Makerere University Research and Ethics Committee (Kampala, Uganda), the Uganda National Council on Science and Technology, and the Research Ethics Board of the University Health Network, Toronto, Canada (REB# 10-0607-B). The research is being conducted in accordance with the Declaration of Helsinki.

Informed consent will be obtained from the parent/guardian of all children that participate in the study. Trained study team personnel will seek consent after a comprehensive discussion with the parent/guardian of a prospective participant in the local language (Lusoga) at an education-appropriate level. Assent will also be sought from those children who are alert and able to understand the trial (age 8 and above), and sustained dissent on the part of children will be honoured [57]. Specific aspects included in the consent discussion include: the acuity and potential lethality of severe malaria, residual mortality despite antiparasitic treatment, the absence of proven effective adjunctive therapies, potential benefits and harms of iNO, the concept of randomization and potential allocation to placebo control (although all patients will receive standard care including potent antimalarials), blinding of treatment allocation, blood samples required for the trial above what is necessary for clinical care, and the distinction between the experimental intervention and clinical care.

International collaborative research may face complex community challenges [58,59]. Community engagement (CE), a participatory process of collaboration and exchange between the various key stakeholders in the research process, may mitigate risks with respect to trial success, optimize participant retention, and minimize social disruption by providing a platform to seek input from and provide ongoing feedback to community members [60]. There is currently no consensus on what CE activities are required in clinical trials, but a recently published model of CE in global health research provides a useful framework of key CE activities and their ethical implications [60].

We will use CE to improve awareness of our trial and its findings in the catchment area of the Jinja Regional Referral Hospital. We also hope the CE activities will contribute to key ethical objectives for the trial, including respect for communities, fairness, transparency and accountability of the trial overall. We will focus on the following CE activities and their associated aims: (1) understanding the relevant community by consciously reaching out beyond the hospital to its catchment areas and listening to their issues and concerns; (2) providing information about the trial, including the pre-clinical evidence behind iNO, timeframe, procedures, and what will happen if the trial is successful; (3) building relationships and trust with local frontline healthcare workers; (4) specific educational/training activities, based on consultation with the nurses and/or frontline healthcare workers to ascertain what would be most relevant and beneficial for them; and (5) feedback of trial results, guided by the community itself as to how and what types of feedback activities would be most appropriate. Several levels of community will be targeted, including parents and primary caregivers of children, who comprise the group at highest risk of malaria, as well as healthcare professionals within the hospital catchment area. These activities do not constitute a mechanism for recruitment of participants to the trial, since only children with severe malaria will be eligible. Instead the community engagement process is intended to build trust and avoid misunderstandings through a dynamic exchange of information and ideas between trial scientists and community members.

### Sample Size Calculation

We will enrol 180 patients (approximately 90 in each treatment arm). To arrive at this sample size estimate, we began with a preliminary calculation based on data from a recent clinical data in severe malaria, in which Ang-2 decreased by 2700 pg/mL/day (95% CI 1800-3600 pg/mL/day) [28]. We assume that a 50% change in this parameter would represent a clinically significant therapeutic effect. By standard calculations for normally distributed data, 80 patients per group will provide 80% power to detect a difference between two treatment arms of 1350 pg/mL/day at p = 0.05 (two-sided). To account for possible dropout, loss to follow-up, and/or non-evaluable data of 10% of patients, approximately 90 patients per study arm are required.

To validate this preliminary sample size estimate, consistent with our analytic plan (mixed-effects linear model), a simulation study was performed. A number of assumptions needed to be made, such as within-patient correlation and Ang-2 variability. Patient Ang-2 data were simulated using a multivariate normal distribution. A simple autoregressive correlation structure was used with correlations of 0.75, 0.5 and 0.25 for time lags of 1, 2 and 3 days, respectively. In previous studies, variability appears proportional to the mean [28]. Simulations were run with three different relationships where the standard deviation was taken to be 40%, 50% and 80% of the Ang-2 mean at each time point. Both groups were assumed to start at Ang-2 levels of 15,000 pg/mL [28] and the average values at each of days 1, 2 and 3 were based on the hypothesized slopes of Ang-2. One thousand replications were performed at each standard deviation relationship and treatment effect. The mixed effects models were fit and the likelihood ratio test was used to test the hypothesis of no *time-by-treatment *interaction at the 5% level (two-tailed). Table 1 shows the results of the power simulations.

**Table 1:** Statistical power (1-β) of a trial comparing nitric oxide with placebo (n = 90 patients per group) for the adjunctive therapy of severe malaria, using longitudinal Ang-2 levels as primary outcome

Standard Deviation of Ang-2 levels at admission (% of mean)	Difference in rate of change of Ang-2 between groups		
	
	30% change (810 pg/mL/day)	40% change (1080 pg/mL/day)	50% change (1350 pg/mL/day)
**40**	82%	97%	99%

**50**	66%	89%	98%

**80**	42%	60%	80%

### Interim Analysis

An interim analysis for efficacy, safety and trial quality is planned at the midpoint of patient enrolment (approximately 45 patients per group). There is no plan to stop the trial prematurely for efficacy or futility based on the primary endpoint (Ang-2), other biochemical parameters, clinical recovery times or parasitological outcomes, given the modest size of the trial and limited statistical power at the midpoint. Data at the time of the interim analysis will be presented to the DSMB members for review, who may advise that the trial continue without modification, continue with changes to the protocol, or be discontinued prematurely. With respect to the statistical interpretation of safety data, the DSMB may recommend termination or modification of the trial if mortality rates exceed statistical thresholds as described above. However, we do not propose that the DSMB be strictly bound by pre-specified criteria, because of the complexity of the trade-offs between safety, efficacy, and the possibility that new information will change considerations. Rather, consideration of stopping guidelines requires a reasoned judgment based on all information that is available at the time of data review.

### Primary Analysis

The primary focus is whether or not the rate of reduction in Ang-2 differs between the treatment groups. In statistical terms, this is a *time-by-treatment *interaction. Given that we will have repeated measurements, possibly incomplete, over time, a linear mixed-effects model will be used to estimate and test the magnitude of the *time-by-treatment *interaction. A linear time trend will not be assumed by treating "day" as a categorical variable in the model. The primary analysis will be by *intention-to-treat *(ITT). That is, patients will be analysed in the group to which they were randomized, regardless of deviations from study protocol. A secondary *per-protocol *analysis may be considered if important deviations from the protocol compromise the validity of the ITT analysis.

### Secondary outcomes

#### Mortality

Analysis will follow standard methods in other clinical trials for malaria [4,48,49]. Mortality at 48 h and 14 days will be coded as a binary variable. Absolute and relative risk reduction will be reported with binomial 95% confidence intervals. Analysis will be by χ^2 ^test or Fisher's exact test. In addition, we will present Kaplan-Meier survival curves comparing patients treated with iNO and placebo. Time to death will be analysed using survival analysis (log-rank test for difference between treatment arms).

#### Time to recovery

Among survivors (a subgroup of randomized participants), recovery times will be analysed by survival analysis (log-rank test). Time to sit unsupported, time to coma resolution (in the subset of patients with coma at study admission) and time to discharge will be documented by treating clinicians blinded to treatment allocation. Time to fever resolution, defined as the time required to achieve a temperature <38°C and the time to maintenance of temperature <38°C, will be determined from frequent vital sign monitoring. The time required to achieve a reduction in parasite density of 50%, 90% and to undetectable levels will be determined from daily blood smears. Results will be expressed as the median time to each event, with 95% confidence intervals.

*Additional biomarkers *(continuous variables, repeated longitudinal measurements) will be analysed using mixed-effects linear models, with the raw value or log-transformed value of the biomarker level as the dependent variable, as appropriate.

#### Neurocognitive outcome

As in previous studies involving children in sub-Saharan Africa [2], standardized instruments will be used for neurocognitive testing: Kaufman Assessment Battery for Children (working memory), the visual form of the computerized Test of Variables of Attention (executive attention), and the Tactual Performance Test (tactile-based learning). Summary variables from each test, converted to age-specific standardized (z) scores, will provide quantitative measures of these three cognitive domains (working memory, attention and tactile learning). Details of these tests are described elsewhere [2,61]. Frequencies of overall cognitive and neurologic deficits in children treated with iNO and children receiving placebo will be compared by χ^2 ^test or Fisher's exact test. Differences in cognitive areas affected in the two age groups (18 mo-4 years, 5-10 years) will be assessed by comparing frequency of individuals with deficits in each area by χ^2 ^test or Fisher's exact test. This will also serve as the best surrogate of impairment in a particular area in one age group versus another, since the type of testing for each cognitive area will be different for the two age groups, so no direct comparison of level of impairment will be possible across age groups. For individual tests, age-adjusted z-scores (determined from normative data in previous studies among healthy community controls) will be analyzed by means of mixed-effects models to examine study group differences in relation to neurocognitive outcomes. The models will provide estimated mean differences between children treated with iNO to placebo controls.

### Subgroup Analyses

Although our sample size is modest, we will explore subgroup effects by examining interaction terms in the mixed-effects linear model. Subgroup analyses related to important prognostic factors will be performed: age < 5 years or ≥5 years; HIV seropositivity; and bacterial co-infection.

### Quality management

Quality management (QM), both quality control and assurance, is a continuous, ongoing process of evaluation of the quality of the conduct and documentation of studies. The first step in QM will be training/re-training of the research staff to ensure consistency in clinical management, sample processing and data collection. Standard operating procedures (SOPs) have been developed for all study related procedures and protocols, and study personnel will document any deviation from SOPs together with the reason for the deviation. The next step for QM will be monitoring of collected data on a prospective basis, with daily review of source documents for completeness, accuracy and consistency. Next, data entry will be verified periodically, and discrepancies will be reviewed with the nurses and medical officers to discover the reason for errors, take corrective measures and prevent future errors. Collection, storage and transport of clinical samples will also be monitored on a regular basis. The ethical conduct of the study will be monitored through initial training in research ethics for study staff, and documentation, mediation and resolution of any perceived violations of ethical standards by participants, their parents/guardians, or members of the community at large. Measures to minimize bias in the trial will be subjected to formal evaluation. Quality of randomization and allocation concealment will be evaluated by keeping sequentially numbered opaque envelopes containing the randomization code, which will be opened, signed and dated at the time of randomization. Quality of trial blinding will be evaluated by asking key trial persons (participants, parents/guardians, medical officers, and nurses) to guess patients' treatments at the end of their trial participation, and compare the answers with the actual treatments, as previously described [62]. External independent oversight of trial quality will be performed by the DSMB, who will review trial quality indices periodically, as well as an external trial auditor.

## Discussion

Nitric oxide is an attractive candidate for an adjunctive therapeutic agent for severe malaria given pre-clinical data on its efficacy in animal models and an established track record of safety in clinical practice and previous trials. Unlike other NO donor molecules, iNO has not been reported to cause systemic vasodilation and hypotension [38]. Furthermore, unlike the NO precursor L-arginine, iNO does not require functional endothelial cell NOS, which may be compromised in patients with severe disease. It is routinely used in clinical practice as an approved agent for hypoxic respiratory failure in neonates, and has an established track record of safety in critically ill patient populations. NO has been used in a wide variety of clinical settings in older children and adults including acute respiratory distress syndrome, pulmonary hypertension, and pregnancy-induced hypertension [63].

Outside the existing patent, iNO is relatively inexpensive [64], and can feasibly delivered by mask [47] in areas with minimal health infrastructure. As currently marketed, INOmax from INO Therapeutics is cost-effective in high-income countries for the treatment of respiratory failure in neonates [65], but may be prohibitively expensive in low- or middle-income countries. The real cost of iNO (not just the price from a single company) is much lower (medical grade iNO $1.99/h compared to $125/h for INOmax) [66]. The patent for INOmax expires in 2013, leaving open the possibility for cheap manufacturing and commercialization of iNO in low-income settings, should iNO prove to be beneficial in severe malaria.

Other adjunctive treatment strategies for severe malaria previously tested in randomized controlled trials include immunomodulation, iron chelation, reduction of oxidative stress, anti-coagulation, volume expansion, reduction of intracranial pressure, and prevention of seizure activity [6]. Only one agent (albumin) was associated with a mortality benefit [6,67,68], although poor methodologic quality in some of these trials may have limited their ability to detect meaningful treatment effects [69]. The search for an effective adjuvant in severe malaria remains a worthwhile goal given the significant residual mortality with primary antiparasitic treatment [4,5]. In this context, iNO, if demonstrated to be effective, would represent an important advance in malaria therapeutics.

Our hypothesis that children with severe malaria will benefit from adjunctive iNO can be answered using a modest sample size (n = 180). This parsimonious design was made possible by selecting a quantitative biomarker of malaria severity, Ang-2, as the primary endpoint and by using a powerful statistical technique (mixed-effects linear model). In contrast, a study using mortality (dichotomous variable) as the primary endpoint would require a total of over 1,000 patients to detect a 30% reduction from the baseline mortality of 20% with 80% power, likely necessitating multicentre recruitment over several years and monumental resources. Ang-2 is a surrogate but well validated measure of malaria severity, appropriate for an early efficacy trial in a human population. Repeated longitudinal measurement of Ang-2 allows for increased precision in the quantitative outcome variable, thereby reducing the necessary sample size. The analytic plan, a mixed-effects linear statistical model, includes random-effect terms, appropriate for representing clustered and therefore dependent data arising when data are gathered over time on the same individuals [70]. We performed a computer simulation study to validate our sample size estimate using assumptions based on previous studies of Ang-2 in severe malaria. In 9,000 simulated trial outcomes under different assumptions for the treatment effect size and baseline variability in Ang-2, the mixed-effects linear model detected a significant treatment effect with >80% power under most plausible scenarios. This *a priori *power calculation provides further refinement on a crude sample size calculation and provides additional evidence that the planned number of patients is adequate to test our hypothesis. This sample size validation is particularly important in light of a review highlighting that numerous previous trials of adjunctive treatments for cerebral malaria had insufficient statistical power to detect even large treatment effects [69].

Some unique aspects of our trial relate to its setting in a peripheral, resource-constrained pediatric hospital in sub-Saharan Africa. Standard clinical investigations including quality-controlled microscopy, biochemistry and microbiology, as well as equipment to monitor gas delivery (NO, NO_2 _and methemoglobin monitoring) need to be introduced to the facility for the trial. A commercially available portable biochemistry instrument (i-STAT^®^, Abbott Point of Care Inc., Princeton, NJ) and a pulse CO-oximeter for non-invasive methemoglobin monitoring (Masimo Rad-57™, Masimo Corporation, Irvine, CA) will allow for onsite monitoring of critical investigations, with outsourcing of other clinical testing to reference laboratories in Kampala. Objective qualitative determination of parasitemia at presentation for the purposes of trial enrolment using commercially available lateral flow immunochromatographic tests for parasite antigen detection (rapid diagnostic tests) will be used to supplement local microscopy, which may be subject to error in the absence of rigorous laboratory quality control [71]. While upgrading hospital capacity for our trial requires the infusion of considerable resources, it is hoped that this will result sustained local capacity development in clinical management, laboratory diagnostics, modern therapeutics, and innovation at the Jinja Regional Referral Hospital, consistent with the ethical objectives of our trial.

The cross-cultural setting of the trial poses certain ethical challenges, demanding a sensitive approach to the informed consent process. While some of these ethical considerations are of a universal nature, others may be more specific to the sub-Saharan African context. First, the trial involves a vulnerable pediatric population with surrogate decision-makers. In addition to parent/guardian consent in the local and education-appropriate language, we have built in a second assent process for competent participants, given that children may legitimately and autonomously participate in decisions related to their own healthcare [57,72]. Next, the acuity and lethality of the underlying infection demand that consent be obtained early after admission for maximal treatment benefit, yet must not interfere with the emergency management of critically ill participants and must give adequate time for stressed parents/guardians to give due consideration before consenting. The complexity of the scientific design (randomized, placebo-controlled adjunctive therapy, with blinding of treatment allocation) together with variable education level and familiarity with biomedical research of parents/guardians presents difficulties requiring careful explanation during the consent discussion (e.g., conveying the position of scientific equipoise in order to accept possible randomization to placebo). Additional ethical considerations which may be more specific to the African context include the "therapeutic misconception," (participants may not clearly distinguish research from health interventions) [73,74]. Furthermore, African parents frequently express concern about blood taking, including fears about the misuse of the blood, unauthorized testing for HIV, long-term storage, genetic testing, and harm to the child from excessive blood loss [73]. Our consent process explicitly addresses the distinction between study and clinical interventions, as well as detailed descriptions of volumes, frequency and subsequent handling of blood samples in the trial. Finally, we have incorporated a community engagement (CE) plan into our trial design to foster a trusting relationship with the surrounding catchment population.

Monitoring patient safety in a trial involving critically ill children in a resource-limited environment poses additional challenges. The administration of study gas will be tightly regulated and monitored with state-of-the-art technology, with particular attention to two dose-dependent, reversible adverse effects: elevated inspired NO_2 _and methemoglobinemia. Strict criteria for study gas discontinuation have been established. Standardized pediatric toxicity tables will be used to monitor for other adverse events in a blinded and objective manner. We will also monitor mortality using statistical control charts, in order to rationally detect deviations from the expected baseline mortality of 20% [42,75]. This approach involves striking a balance between the earliest detection of elevated mortality in order to institute corrective measures ("true alarm"), and the risk of halting the trial unnecessarily for variations in mortality due to chance alone ("false alarm"). The upper control limit of mortality that will prompt a safety review has thus been defined using statistical principles, together with the predicted performance of this surveillance strategy (Additional File 1). Trial safety oversight will ultimately be provided by an independent DSMB who will meet regularly to review recruitment, interim evidence of efficacy, safety and trial quality.

Limitations of this trial design include its use of a surrogate marker, Ang-2, as the primary endpoint. This allows for an efficient design but may be less compelling to clinicians than would a demonstrable mortality benefit (of note, mortality has been included as a secondary trial endpoint). On the other hand, Ang-2 has been extensively validated as an objective and quantitative biomarker of malaria severity [28,29,76,77]. Thus, Ang-2 is an appropriate endpoint for an initial efficacy study of iNO in a human population, but a promising treatment effect would require validation in costly, large, multicentre trials. Blinding presents a challenge for a gaseous therapy requiring monitoring for dose-dependent toxicities, which we have addressed by separating study tasks between a blinded team (responsible for clinical care, assessment of endpoints and laboratory testing) and an unblinded team (responsible for administering treatment and monitoring safety parameters) to minimize potential bias. Risks and costs related to conducting the study in a resource-constrained setting are balanced with potential benefits in terms of local capacity building, the high incidence of severe malaria allowing timely completion of the study, and applicability of the therapy in other malaria-endemic areas.

In summary, based on compelling data supporting the efficacy of iNO in experimental cerebral malaria in animal models, coupled with the documented safety of iNO in clinical practice and trials for other diseases, we have outlined a protocol for a randomized clinical trial of iNO for the adjunctive treatment of severe malaria in Ugandan children. If our study demonstrates a significant treatment effect, this would represent a major and important advance in the treatment of severe malaria with broad potential for global public health impact.

## List of abbreviations

Ang-1: Angiopoietin-1; Ang-2: Angiopoietin-2; CE: community engagement; CONSORT: Consolidated Standards of Reporting Trials; DPTA/NO: dipropylenetriamine NONOate; DSMB: Data and Safety Monitoring Board; ECMO: extracorporeal membrane oxygenation; HRP-2: histidine-rich protein-2; ICAM-1: intercellular cell adhesion molecule-1; iNO: inhaled nitric oxide; NO: nitric oxide; NO_2_^:^nitrogen dioxide; NOS: nitric oxide synthase; PE: parasitized erythrocyte; pLDH: parasite lactate dehydrogenase; QM: quality management; RDT: rapid diagnostic test; sGC: soluble guanylate cyclise; SMA: severe malarial anemia; SOP: standard operating procedure; vWF: von Willebrand Factor; WPB: Weibel-Palade bodies.

## Competing interests

CM is Chief Scientific Officer of Nitric Solutions Inc., developer of nitric oxide (NO) based medical products.

KCK, WCL, and ALC are listed as inventors on a patent owned by University Health Network (Toronto) related to Ang-2 as a biomarker for infectious diseases that compromise endothelial integrity.

All other authors: no conflicts.

## Authors' contributions

MH designed the study and wrote the manuscript. ROO participated in study design and logistical planning, and obtained ethical approval from the Ugandan bodies. SN participated in study design and logistical planning. CM participated in the study design and provided expertise related to iNO administration and safety monitoring. KET provided statistical input, designed the primary statistical analysis, and performed the computer simulation experiment for validation of the sample size. JVL provided expertise in ethics and community engagement. ALC participated in the study design and helped to draft the manuscript. WCL participated in the study design and helped to draft the manuscript. CCJ participated in the study design and helped to draft the manuscript. KCK conceived the study, participated in the study design and helped to draft the manuscript. All authors read and approved the final manuscript.

## Supplementary Material

Additional file 1 **Details of a process control chart to monitor mortality**. This Microsoft Word file provides details of the statistical thresholds and the performance characteristics of a statistical control chart to monitor mortality in this trial of critically ill children. Click here for file

## References

[B1] WHO World malaria report 2008 2008 WHO, Geneva, Switzerland

[B2] John CC Bangirana P Byarugaba J Opoka RO Idro R Jurek AM Wu B Boivin MJ Cerebral malaria in children is associated with long-term cognitive impairment Pediatrics 2008 122 e92 99 10.1542/peds.2007-3709 18541616 PMC2607241

[B3] Newton CR Krishna S Severe falciparum malaria in children: current understanding of pathophysiology and supportive treatment Pharmacol Ther 1998 79 1 53 10.1016/S0163-7258(98)00008-4 9719344

[B4] Dondorp A Nosten F Stepniewska K Day N White N Artesunate versus quinine for treatment of severe falciparum malaria: a randomised trial Lancet 2005 366 717 725 16125588 10.1016/S0140-6736(05)67176-0

[B5] Dondorp AM Fanello CI Hendriksen IC Gomes E Seni A Chhaganlal KD Bojang K Olaosebikan R Anunobi N Maitland K Artesunate versus quinine in the treatment of severe falciparum malaria in African children (AQUAMAT): an open-label, randomised trial Lancet 376 1647 1657 10.1016/S0140-6736(10)61924-1 PMC3033534 21062666

[B6] John CC Kutamba E Mugarura K Opoka RO Adjunctive therapy for cerebral malaria and other severe forms of Plasmodium falciparum malaria Expert Rev Anti Infect Ther 8 997 1008 10.1586/eri.10.90 PMC2987235 20818944

[B7] Korhonen R Lahti A Kankaanranta H Moilanen E Nitric oxide production and signaling in inflammation Curr Drug Targets Inflamm Allergy 2005 4 471 479 10.2174/1568010054526359 16101524

[B8] Bogdan C Nitric oxide and the immune response Nat Immunol 2001 2 907 916 11577346 10.1038/ni1001-907

[B9] Matsushita K Morrell CN Cambien B Yang SX Yamakuchi M Bao C Hara MR Quick RA Cao W O'Rourke B Nitric oxide regulates exocytosis by S-nitrosylation of N-ethylmaleimide-sensitive factor Cell 2003 115 139 150 10.1016/S0092-8674(03)00803-1 14567912 PMC2846406

[B10] Palmer RM Ferrige AG Moncada S Nitric oxide release accounts for the biological activity of endothelium-derived relaxing factor Nature 1987 327 524 526 10.1038/327524a0 3495737

[B11] Gramaglia I Sobolewski P Meays D Contreras R Nolan JP Frangos JA Intaglietta M van der Heyde HC Low nitric oxide bioavailability contributes to the genesis of experimental cerebral malaria Nat Med 2006 12 1417 1422 17099710 10.1038/nm1499

[B12] Anstey NM Weinberg JB Hassanali MY Mwaikambo ED Manyenga D Misukonis MA Arnelle DR Hollis D McDonald MI Granger DL Nitric oxide in Tanzanian children with malaria: inverse relationship between malaria severity and nitric oxide production/nitric oxide synthase type 2 expression J Exp Med 1996 184 557 567 10.1084/jem.184.2.557 8760809 PMC2192721

[B13] Lopansri BK Anstey NM Weinberg JB Stoddard GJ Hobbs MR Levesque MC Mwaikambo ED Granger DL Low plasma arginine concentrations in children with cerebral malaria and decreased nitric oxide production Lancet 2003 361 676 678 10.1016/S0140-6736(03)12564-0 12606182

[B14] Yeo TW Lampah DA Tjitra E Gitawati R Darcy CJ Jones C Kenangalem E McNeil YR Granger DL Lopansri BK Increased asymmetric dimethylarginine in severe falciparum malaria: association with impaired nitric oxide bioavailability and fatal outcome PLoS Pathog 6 e1000868 10.1371/journal.ppat.1000868 PMC2858698 20421938

[B15] Kun JF Mordmuller B Perkins DJ May J Mercereau-Puijalon O Alpers M Weinberg JB Kremsner PG Nitric oxide synthase 2(Lambarene) (G-954C), increased nitric oxide production, and protection against malaria J Infect Dis 2001 184 330 336 10.1086/322037 11443559

[B16] Dhangadamajhi G Mohapatra BN Kar SK Ranjit MR The CCTTT pentanucleotide microsatellite in iNOS promoter influences the clinical outcome in P. falciparum infection Parasitol Res 2009 104 1315 1320 10.1007/s00436-009-1329-9 19153766

[B17] Boutlis CS Hobbs MR Marsh RL Misukonis MA Tkachuk AN Lagog M Booth J Granger DL Bockarie MJ Mgone CS Inducible nitric oxide synthase (NOS2) promoter CCTTT repeat polymorphism: relationship to in vivo nitric oxide production/NOS activity in an asymptomatic malaria-endemic population Am J Trop Med Hyg 2003 69 569 573 14740870

[B18] Ohashi J Naka I Patarapotikul J Hananantachai H Looareesuwan S Tokunaga K Significant association of longer forms of CCTTT Microsatellite repeat in the inducible nitric oxide synthase promoter with severe malaria in Thailand J Infect Dis 2002 186 578 581 10.1086/341779 12195390

[B19] Kun JF Mordmuller B Lell B Lehman LG Luckner D Kremsner PG Polymorphism in promoter region of inducible nitric oxide synthase gene and protection against malaria Lancet 1998 351 265 266 10.1016/S0140-6736(05)78273-8 9457101

[B20] Sobolewski P Gramaglia I Frangos J Intaglietta M van der Heyde HC Nitric oxide bioavailability in malaria Trends Parasitol 2005 21 415 422 10.1016/j.pt.2005.07.002 16039159

[B21] Day NP Phu NH Mai NT Chau TT Loc PP Chuong LV Sinh DX Holloway P Hien TT White NJ The pathophysiologic and prognostic significance of acidosis in severe adult malaria Crit Care Med 2000 28 1833 1840 10.1097/00003246-200006000-00025 10890629

[B22] MacPherson GG Warrell MJ White NJ Looareesuwan S Warrell DA Human cerebral malaria. A quantitative ultrastructural analysis of parasitized erythrocyte sequestration Am J Pathol 1985 119 385 401 3893148 PMC1888001

[B23] Turner GD Morrison H Jones M Davis TM Looareesuwan S Buley ID Gatter KC Newbold CI Pukritayakamee S Nagachinta B An immunohistochemical study of the pathology of fatal malaria. Evidence for widespread endothelial activation and a potential role for intercellular adhesion molecule-1 in cerebral sequestration Am J Pathol 1994 145 1057 1069 7526692 PMC1887431

[B24] De Caterina R Libby P Peng HB Thannickal VJ Rajavashisth TB Gimbrone MA Jr Shin WS Liao JK Nitric oxide decreases cytokine-induced endothelial activation. Nitric oxide selectively reduces endothelial expression of adhesion molecules and proinflammatory cytokines J Clin Invest 1995 96 60 68 10.1172/JCI118074 7542286 PMC185173

[B25] Jakobsen PH Morris-Jones S Ronn A Hviid L Theander TG Elhassan IM Bygbjerg IC Greenwood BM Increased plasma concentrations of sICAM-1, sVCAM-1 and sELAM-1 in patients with Plasmodium falciparum or P. vivax malaria and association with disease severity Immunology 1994 83 665 669 7533138 PMC1415057

[B26] de Mast Q Groot E Lenting PJ de Groot PG McCall M Sauerwein RW Fijnheer R van der Ven A Thrombocytopenia and release of activated von Willebrand Factor during early Plasmodium falciparum malaria J Infect Dis 2007 196 622 628 10.1086/519844 17624850

[B27] Hollestelle MJ Donkor C Mantey EA Chakravorty SJ Craig A Akoto AO O'Donnell J van Mourik JA Bunn J von Willebrand factor propeptide in malaria: evidence of acute endothelial cell activation Br J Haematol 2006 133 562 569 10.1111/j.1365-2141.2006.06067.x 16681646

[B28] Yeo TW Lampah DA Gitawati R Tjitra E Kenangalem E Piera K Price RN Duffull SB Celermajer DS Anstey NM Angiopoietin-2 is associated with decreased endothelial nitric oxide and poor clinical outcome in severe falciparum malaria Proc Natl Acad Sci USA 2008 105 17097 17102 10.1073/pnas.0805782105 18957536 PMC2575222

[B29] Lovegrove FE Tangpukdee N Opoka RO Lafferty EI Rajwans N Hawkes M Krudsood S Looareesuwan S John CC Liles WC Kain KC Serum angiopoietin-1 and -2 levels discriminate cerebral malaria from uncomplicated malaria and predict clinical outcome in African children PLoS One 2009 4 e4912 10.1371/journal.pone.0004912 19300530 PMC2657207

[B30] Bridges DJ Bunn J van Mourik JA Grau G Preston RJ Molyneux M Combes V O'Donnell JS de Laat B Craig A Rapid activation of endothelial cells enables Plasmodium falciparum adhesion to platelet-decorated von Willebrand factor strings Blood 2010 115 1472 1474 10.1182/blood-2009-07-235150 19897581 PMC2840836

[B31] Fiedler U Reiss Y Scharpfenecker M Grunow V Koidl S Thurston G Gale NW Witzenrath M Rosseau S Suttorp N Angiopoietin-2 sensitizes endothelial cells to TNF-alpha and has a crucial role in the induction of inflammation Nat Med 2006 12 235 239 10.1038/nm1351 16462802

[B32] Parikh SM Mammoto T Schultz A Yuan HT Christiani D Karumanchi SA Sukhatme VP Excess circulating angiopoietin-2 may contribute to pulmonary vascular leak in sepsis in humans PLoS Med 2006 3 e46 10.1371/journal.pmed.0030046 16417407 PMC1334221

[B33] Saharinen P Eklund L Miettinen J Wirkkala R Anisimov A Winderlich M Nottebaum A Vestweber D Deutsch U Koh GY Angiopoietins assemble distinct Tie2 signalling complexes in endothelial cell-cell and cell-matrix contacts Nat Cell Biol 2008 10 527 537 10.1038/ncb1715 18425119

[B34] Thurston G Suri C Smith K McClain J Sato TN Yancopoulos GD McDonald DM Leakage-resistant blood vessels in mice transgenically overexpressing angiopoietin-1 Science 1999 286 2511 2514 10.1126/science.286.5449.2511 10617467

[B35] Fukuhara S Sako K Minami T Noda K Kim HZ Kodama T Shibuya M Takakura N Koh GY Mochizuki N Differential function of Tie2 at cell-cell contacts and cell-substratum contacts regulated by angiopoietin-1 Nat Cell Biol 2008 10 513 526 10.1038/ncb1714 18425120

[B36] Yeo TW Lampah DA Gitawati R Tjitra E Kenangalem E McNeil YR Darcy CJ Granger DL Weinberg JB Lopansri BK Impaired nitric oxide bioavailability and L-arginine reversible endothelial dysfunction in adults with falciparum malaria J Exp Med 2007 204 2693 2704 10.1084/jem.20070819 17954570 PMC2118490

[B37] Finer NN Barrington KJ Nitric oxide for respiratory failure in infants born at or near term Cochrane Database Syst Rev 2006 CD000399 10.1002/14651858.CD000399.pub2 17054129

[B38] Adhikari NK Burns KE Friedrich JO Granton JT Cook DJ Meade MO Effect of nitric oxide on oxygenation and mortality in acute lung injury: systematic review and meta-analysis BMJ 2007 334 779 10.1136/bmj.39139.716794.55 17383982 PMC1852043

[B39] Schulz KF Altman DG Moher D CONSORT 2010 statement: updated guidelines for reporting parallel group randomised trials Bmj 340 c332 10.1136/bmj.c332 PMC2844940 20332509

[B40] WHO Malaria rapid diagnostic test performance: results of WHO product testing of malaria RDTs: round 1 2008 Geneva, Switzerland: WHO

[B41] WHO Management of severe malnutrition: a manual for physicians and other senior health workers 1999 Geneva, Switzerland: WHO

[B42] Idro R Aloyo J Mayende L Bitarakwate E John CC Kivumbi GW Severe malaria in children in areas with low, moderate and high transmission intensity in Uganda Trop Med Int Health 2006 11 115 124 10.1111/j.1365-3156.2005.01518.x 16398762

[B43] Inhaled nitric oxide in full-term and nearly full-term infants with hypoxic respiratory failure. The Neonatal Inhaled Nitric Oxide Study Group N Engl J Med 1997 336 597 604 9036320 10.1056/NEJM199702273360901

[B44] Davidson D Barefield ES Kattwinkel J Dudell G Damask M Straube R Rhines J Chang CT Inhaled nitric oxide for the early treatment of persistent pulmonary hypertension of the term newborn: a randomized, double-masked, placebo-controlled, dose-response, multicenter study. The I-NO/PPHN Study Group Pediatrics 1998 101 325 334 10.1542/peds.101.3.325 9480993

[B45] Head CA Swerdlow P McDade WA Joshi RM Ikuta T Cooper ML Eckman JR Beneficial effects of nitric oxide breathing in adult patients with sickle cell crisis Am J Hematol 85 800 802 10.1002/ajh.21832 20799359

[B46] Weiner DL Hibberd PL Betit P Cooper AB Botelho CA Brugnara C Preliminary assessment of inhaled nitric oxide for acute vaso-occlusive crisis in pediatric patients with sickle cell disease Jama 2003 289 1136 1142 10.1001/jama.289.9.1136 12622584

[B47] Long R Jones R Talbot J Mayers I Barrie J Hoskinson M Light B Inhaled nitric oxide treatment of patients with pulmonary tuberculosis evidenced by positive sputum smears Antimicrob Agents Chemother 2005 49 1209 1212 10.1128/AAC.49.3.1209-1212.2005 15728930 PMC549277

[B48] Tran TH Day NP Nguyen HP Nguyen TH Pham PL Dinh XS Ly VC Ha V Waller D Peto TE White NJ A controlled trial of artemether or quinine in Vietnamese adults with severe falciparum malaria N Engl J Med 1996 335 76 83 10.1056/NEJM199607113350202 8649493

[B49] van Hensbroek MB Onyiorah E Jaffar S Schneider G Palmer A Frenkel J Enwere G Forck S Nusmeijer A Bennett S A trial of artemether or quinine in children with cerebral malaria N Engl J Med 1996 335 69 75 10.1056/NEJM199607113350201 8649492

[B50] Troncy E Collet JP Shapiro S Guimond JG Blair L Ducruet T Francoeur M Charbonneau M Blaise G Inhaled nitric oxide in acute respiratory distress syndrome: a pilot randomized controlled study Am J Respir Crit Care Med 1998 157 1483 1488 9603127 10.1164/ajrccm.157.5.9707090

[B51] Griffiths MJ Evans TW Inhaled nitric oxide therapy in adults N Engl J Med 2005 353 2683 2695 10.1056/NEJMra051884 16371634

[B52] Leavey JF Dubin RL Singh N Kaminsky DA Silo-Filler's disease, the acute respiratory distress syndrome, and oxides of nitrogen Ann Intern Med 2004 141 410 411 15353441 10.7326/0003-4819-141-5-200409070-00031

[B53] Elsayed NM Toxicity of nitrogen dioxide: an introduction Toxicology 1994 89 161 174 10.1016/0300-483X(94)90096-5 8023327

[B54] Svolba G Bauer P Statistical quality control in clinical trials Control Clin Trials 1999 20 519 530 10.1016/S0197-2456(99)00029-X 10588293

[B55] NIAID Division of Microbiology and Infectious Diseases Pediatric Toxicity Tables (revised 11.21.07)

[B56] WHO WHO standard definitions for adverse event http://www.who.int/vaccines-documents/DocsPDF05/815.pdf.

[B57] Wendler DS Assent in paediatric research: theoretical and practical considerations J Med Ethics 2006 32 229 234 10.1136/jme.2004.011114 16574878 PMC2588342

[B58] Newman PA Towards a science of community engagement Lancet 2006 367 302 16443036 10.1016/S0140-6736(06)68067-7

[B59] Tindana PO Singh JA Tracy CS Upshur RE Daar AS Singer PA Frohlich J Lavery JV Grand challenges in global health: community engagement in research in developing countries PLoS Med 2007 4 e273 10.1371/journal.pmed.0040273 17850178 PMC1989740

[B60] Lavery JV Tinadana PO Scott TW Harrington LC Ramsey JM Ytuarte-Nunez C James AA Towards a framework for community engagement in global health research Trends Parasitol 26 279 283 10.1016/j.pt.2010.02.009 20299285

[B61] Boivin MJ Bangirana P Byarugaba J Opoka RO Idro R Jurek AM John CC Cognitive impairment after cerebral malaria in children: a prospective study Pediatrics 2007 119 e360 366 10.1542/peds.2006-2027 17224457 PMC2743741

[B62] Hrobjartsson A Forfang E Haahr MT Als-Nielsen B Brorson S Blinded trials taken to the test: an analysis of randomized clinical trials that report tests for the success of blinding Int J Epidemiol 2007 36 654 663 10.1093/ije/dym020 17440024

[B63] Sokol J Jacobs SE Bohn D Inhaled nitric oxide for acute hypoxemic respiratory failure in children and adults Cochrane Database Syst Rev 2003 CD002787 10.1002/14651858.CD002787 12535438

[B64] Hansen TW Inhaled nitric oxide and the societal perspective Pediatrics 2004 113 1849 1851; author reply 1849-1851 10.1542/peds.113.6.1849 15173527

[B65] Angus DC Clermont G Watson RS Linde-Zwirble WT Clark RH Roberts MS Cost-effectiveness of inhaled nitric oxide in the treatment of neonatal respiratory failure in the United States Pediatrics 2003 112 1351 1360 10.1542/peds.112.6.1351 14654609

[B66] Pierce CM Peters MJ Cohen G Goldman AP Petros AJ Cost of nitric oxide is exorbitant Bmj 2002 325 336 10.1136/bmj.325.7359.336 12169517 PMC1123840

[B67] Maitland K Nadel S Pollard AJ Williams TN Newton CR Levin M Management of severe malaria in children: proposed guidelines for the United Kingdom Bmj 2005 331 337 343 10.1136/bmj.331.7512.337 16081449 PMC1183138

[B68] Akech S Gwer S Idro R Fegan G Eziefula AC Newton CR Levin M Maitland K Volume expansion with albumin compared to gelofusine in children with severe malaria: results of a controlled trial PLoS Clin Trials 2006 1 e21 10.1371/journal.pctr.0010021 16998584 PMC1569382

[B69] Enwere G A review of the quality of randomized clinical trials of adjunctive therapy for the treatment of cerebral malaria Trop Med Int Health 2005 10 1171 1175 10.1111/j.1365-3156.2005.01505.x 16262742

[B70] Laird NM Ware JH Random-effects models for longitudinal data Biometrics 1982 38 963 974 10.2307/2529876 7168798

[B71] Hawkes M Kain KC Advances in malaria diagnosis Expert Rev Anti Infect Ther 2007 5 485 495 10.1586/14787210.5.3.485 17547512

[B72] Henschel AD Rothenberger LG Boos J Randomized clinical trials in children--ethical and methodological issues Curr Pharm Des 16 2407 2415 10.2174/138161210791959854 20513232

[B73] Molyneux CS Peshu N Marsh K Trust and informed consent: insights from community members on the Kenyan coast Soc Sci Med 2005 61 1463 1473 10.1016/j.socscimed.2004.11.073 16005781

[B74] Lynoe N Hyder Z Chowdhury M Ekstrom L Obtaining informed consent in Bangladesh N Engl J Med 2001 344 460 461 11221611 10.1056/NEJM200102083440617

[B75] Kyu HH Fernandez E Artemisinin derivatives versus quinine for cerebral malaria in African children: a systematic review Bull World Health Organ 2009 87 896 904 10.2471/BLT.08.060327 20454480 PMC2789363

[B76] Conroy AL Lafferty EI Lovegrove FE Krudsood S Tangpukdee N Liles WC Kain KC Whole blood angiopoietin-1 and -2 levels discriminate cerebral and severe (non-cerebral) malaria from uncomplicated malaria Malar J 2009 8 295 10.1186/1475-2875-8-295 20003529 PMC2806378

[B77] Conroy AL Phiri H Hawkes M Glover S Mallewa M Seydel KB Taylor TE Molyneux ME Kain KC Endothelium-based biomarkers are associated with cerebral malaria in malawian children: a retrospective case-control study PLoS One 5 e15291 10.1371/journal.pone.0015291 PMC3012131 21209923

