# Supplementary material for: Inhaled nitric oxide for the adjunctive therapy of severe malaria: Protocol for a randomized controlled trial
Source: Trials. 2011 Jul 13;12:176. doi: 10.1186/1745-6215-12-176 (PMC3151218; doi:10.1186/1745-6215-12-176)
Supplement: Additional file 1 — Details of a process control chart to monitor mortality. This Microsoft Word file provides details of the statistical thresholds and the performance characteristics of a statistical control chart to monitor mortality in this trial of critically ill children. [file 1745-6215-12-176-S1.DOC]

**Additional file 1 – Details of a process control chart to monitor mortality.**

For each consecutive group of 10 patients (irrespective of group assignment), the mortality will be computed and plotted on the control chart (see Figure, below). Points lying above the upper control limit (UCL) will trigger trial halting, a review of trial procedures for possible causes of elevated mortality, and initiating corrective measures. The probability of a type I error (“false alarm,” halting the trial unnecessarily) and type II error (when the chart fails to indicate the presence of a deviation from baseline mortality) under this scheme can be quantified with some assumptions.

We determined an expected level of mortality from a literature review of clinical trials involving severe malaria in children in sub-Saharan Africa [1]. The mortality in these controlled trials ranged from 6-29%, with a median of 19%. Data from Uganda indicates that the mortality associated with severe malaria is comparable (16% of children with impaired consciousness, and 21% of children with deep acidotic breathing) [2]. The mortality rate observed in the clinical trial is therefore expected to be approximately 20%, with random variation about this baseline value as we sample small groups of patients for monitoring purposes.

The upper control limit was determined using a traditional approach under assumptions that the distribution of mortality will follow a normal approximation to the binomial distribution, and balancing the “detection capacity” (minimizing type II error rate) while minimizing the probability of “false alarms” (type I error rate). This yielded an upper control limit of 57%, or a threshold of 6 deaths in a sample of 10 patients.

An index of the effectiveness of a process control method is the time needed to detect a change after it occurs. As long as a clinical trial runs along an expected level of mortality, the length of the run (the number of inspections for mortality) up to an “out-of-control alarm” should be large. Unnecessary correcting actions, which may be costly and time consuming, should be avoided. Using the process control scheme proposed, if mortality remains constant at 20%, the average run length (ARL) leading to a “false alarm” is 157 inspections (1570 patients), and in our trial of 180 patients, the probability of halting the trial unnecessarily for random variations in the mortality is at most 10.8%. On the other hand, if a significant change occurs in the process, the number of inspections needed to detect it should be as small as possible. This helps initiate actions to investigate the cause of the elevated mortality as quickly as possible. Under the proposed monitoring scheme, if the mortality rate doubles from 20% to 40%, an ARL of 6 inspections (60 patients) will lead to a “true alarm,” prompting halting of the trial and review of procedures for remediable actions.


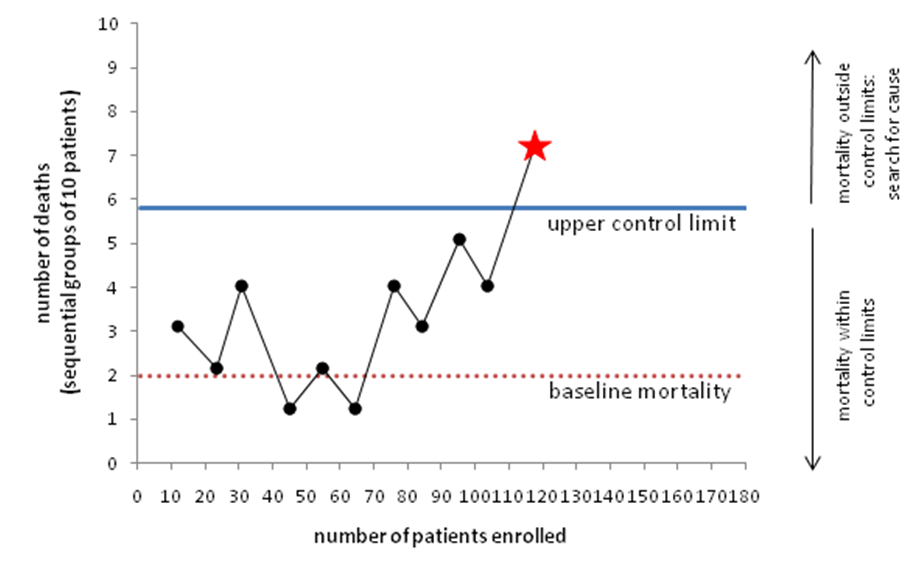


**Figure. Sample control chart for patient mortality.** Mortality is expected in a trial involving critically ill children due to the underlying disease process. We will monitor the mortality rate in our trial using a process control chart for deviations from the expected level (approximately 20%). For sequential groups of 10 patients, the mortality will be plotted, and mortality above the upper control limit will prompt an evaluation for assignable causes in order to institute early corrective measures. Illustrated is an example of a control chart, with mortality outside the control limits detected a group of patients (red star) which would trigger an evaluation of trial safety.

**References**

1. Kyu HH, Fernandez E: **Artemisinin derivatives versus quinine for cerebral malaria in African children: a systematic review.** *Bull World Health Organ* 2009, **87:**896-904.

2. Idro R, Aloyo J, Mayende L, Bitarakwate E, John CC, Kivumbi GW: **Severe malaria in children in areas with low, moderate and high transmission intensity in Uganda.** *Trop Med Int Health* 2006, **11:**115-124.
